# Supplementary material for: The genome sequence of the Eastern Pale Clouded Yellow, Colias erate (Esper, 1805) (Lepidoptera: Pieridae)
Source: Wellcome Open Res. 2026 Feb 9;11:102. [Version 1] doi: 10.12688/wellcomeopenres.25891.1 (PMC13058573; doi:10.12688/wellcomeopenres.25891.1)
Supplement: Peer review history

# Reviewer report: Reviewer response for version 1

**DOI:** 10.21956/wellcomeopenres.28498.r148914

**Published:** 2026-04-07

**Authors:** Xue Qing

**Affiliations:** 1 Nanjing Agricultural University, Nanjing, China

This manuscript presents a high-quality, chromosome-level genome assembly for *Colias erate* . The methods are technically sound, and the assembly meets EBP reference standards. I only have one comment:

 Haplotype 1 is assembled to chromosome level, while Haplotype 2 remains at scaffold level. No explanation is provided for why Hi-C data could not be used to scaffold Haplotype 2 similarly.State whether this was due to coverage, heterozygosity, or Hi-C mapping limitations. If possible, provide a Hi-C contact map or alignment statistics for Haplotype 2 in supplementary materials.

Are sufficient details of methods and materials provided to allow replication by others?

Yes

Is the rationale for creating the dataset(s) clearly described?

Yes

Are the datasets clearly presented in a useable and accessible format?

Yes

Are the protocols appropriate and is the work technically sound?

Yes

Reviewer Expertise:

phylogeny, genomics, Metazoa

I confirm that I have read this submission and believe that I have an appropriate level of expertise to confirm that it is of an acceptable scientific standard.

# Reviewer report: Reviewer response for version 1

**DOI:** 10.21956/wellcomeopenres.28498.r149683

**Published:** 2026-04-02

**Authors:** Emily Hornett

**Affiliations:** 1 University of Liverpool, Liverpool, UK

This manuscript reports the genome of the butterfly *Colias erate*, which was sequenced and assembled as part of Project Psyche, a collaborative programme aiming to sequence the genomes of European butterflies and moths.

 I have two very minor comments for the introduction:

- change "while females have the black border interrupted" to "while females have the black border interrupted with yellow blotches" or similar
- add sex to figure 1 legend (male)

Are sufficient details of methods and materials provided to allow replication by others?

Yes

Is the rationale for creating the dataset(s) clearly described?

Yes

Are the datasets clearly presented in a useable and accessible format?

Yes

Are the protocols appropriate and is the work technically sound?

Yes

Reviewer Expertise:

Insect and microbe genomics, symbiosis, evolution

I confirm that I have read this submission and believe that I have an appropriate level of expertise to confirm that it is of an acceptable scientific standard.
